# Supplementary material for: Pyruvate Dehydrogenase Kinase 1 inhibition mediated oxidative phosphorylation enhancement in cartilage promotes osteoarthritis progression
Source: BMC Musculoskelet Disord. 2023 Jul 20;24:597. doi: 10.1186/s12891-023-06585-6 (PMC10357736; doi:10.1186/s12891-023-06585-6)
Supplement: Supplementary file 1 — Additional file 1. [file 12891_2023_6585_MOESM1_ESM.zip › MF.Gsea.1653627316242/GOMF_PROTEIN_PHOSPHATASE_2A_BINDING.html]

Details for gene set GOMF\_PROTEIN\_PHOSPHATASE\_2A\_BINDING[GSEA]

|  || Dataset | 55457\_collapsed\_to\_symbols.cls.cls #OA\_versus\_normal.cls.cls #OA\_versus\_normal\_repos |
| Phenotype | cls.cls#OA\_versus\_normal\_repos |
| Upregulated in class | normal |
| GeneSet | GOMF\_PROTEIN\_PHOSPHATASE\_2A\_BINDING |
| Enrichment Score (ES) | -0.51605535 |
| Normalized Enrichment Score (NES) | -1.6369852 |
| Nominal p-value | 0.016694492 |
| FDR q-value | 0.1384002 |
| FWER p-Value | 0.981 |
Table: GSEA Results Summary

  

Fig 1: Enrichment plot: GOMF\_PROTEIN\_PHOSPHATASE\_2A\_BINDING      
 Profile of the Running ES Score & Positions of GeneSet Members on the Rank Ordered List

  

| SYMBOL | TITLE | RANK IN GENE LIST | RANK METRIC SCORE | RUNNING ES | CORE ENRICHMENT || 1 | ENSA | endosulfine alpha [Source:HGNC Symbol;Acc:HGNC:3360] | 2172 | 0.341 | -0.1136 | No |
| 2 | IGBP1 | immunoglobulin binding protein 1 [Source:HGNC Symbol;Acc:HGNC:5461] | 3413 | 0.210 | -0.1761 | No |
| 3 | MAPT | microtubule associated protein tau [Source:HGNC Symbol;Acc:HGNC:6893] | 3976 | 0.158 | -0.1942 | No |
| 4 | STAT1 | signal transducer and activator of transcription 1 [Source:HGNC Symbol;Acc:HGNC:11362] | 4495 | 0.113 | -0.2163 | No |
| 5 | TP53 | tumor protein p53 [Source:HGNC Symbol;Acc:HGNC:11998] | 5511 | 0.032 | -0.2900 | No |
| 6 | BCL2 | BCL2 apoptosis regulator [Source:HGNC Symbol;Acc:HGNC:990] | 5609 | 0.023 | -0.2937 | No |
| 7 | PTPA | protein phosphatase 2 phosphatase activator [Source:HGNC Symbol;Acc:HGNC:9308] | 6120 | -0.014 | -0.3311 | No |
| 8 | STRN4 | striatin 4 [Source:HGNC Symbol;Acc:HGNC:15721] | 6507 | -0.043 | -0.3542 | No |
| 9 | FOXO1 | forkhead box O1 [Source:HGNC Symbol;Acc:HGNC:3819] | 6511 | -0.043 | -0.3474 | No |
| 10 | ADCY8 | adenylate cyclase 8 [Source:HGNC Symbol;Acc:HGNC:239] | 6646 | -0.053 | -0.3493 | No |
| 11 | GNA12 | G protein subunit alpha 12 [Source:HGNC Symbol;Acc:HGNC:4380] | 7294 | -0.102 | -0.3832 | No |
| 12 | PPME1 | protein phosphatase methylesterase 1 [Source:HGNC Symbol;Acc:HGNC:30178] | 7483 | -0.117 | -0.3789 | No |
| 13 | PTPN1 | protein tyrosine phosphatase non-receptor type 1 [Source:HGNC Symbol;Acc:HGNC:9642] | 8829 | -0.220 | -0.4479 | No |
| 14 | ARPP19 | cAMP regulated phosphoprotein 19 [Source:HGNC Symbol;Acc:HGNC:16967] | 8912 | -0.225 | -0.4179 | No |
| 15 | SLC6A3 | solute carrier family 6 member 3 [Source:HGNC Symbol;Acc:HGNC:11049] | 10177 | -0.338 | -0.4615 | Yes |
| 16 | SMG5 | SMG5 nonsense mediated mRNA decay factor [Source:HGNC Symbol;Acc:HGNC:24644] | 10190 | -0.340 | -0.4076 | Yes |
| 17 | RPS6KB1 | ribosomal protein S6 kinase B1 [Source:HGNC Symbol;Acc:HGNC:10436] | 10224 | -0.343 | -0.3548 | Yes |
| 18 | SMG7 | SMG7 nonsense mediated mRNA decay factor [Source:HGNC Symbol;Acc:HGNC:16792] | 10877 | -0.414 | -0.3387 | Yes |
| 19 | STRN3 | striatin 3 [Source:HGNC Symbol;Acc:HGNC:15720] | 11457 | -0.497 | -0.3036 | Yes |
| 20 | MFHAS1 | multifunctional ROCO family signaling regulator 1 [Source:HGNC Symbol;Acc:HGNC:16982] | 11898 | -0.571 | -0.2456 | Yes |
| 21 | ANKLE2 | ankyrin repeat and LEM domain containing 2 [Source:HGNC Symbol;Acc:HGNC:29101] | 12043 | -0.604 | -0.1593 | Yes |
| 22 | SPHK1 | sphingosine kinase 1 [Source:HGNC Symbol;Acc:HGNC:11240] | 12320 | -0.682 | -0.0707 | Yes |
| 23 | STRN | striatin [Source:HGNC Symbol;Acc:HGNC:11424] | 12429 | -0.717 | 0.0366 | Yes |
Table: GSEA details [plain text format]

  

Fig 2: GOMF\_PROTEIN\_PHOSPHATASE\_2A\_BINDING      
 Blue-Pink O' Gram in the Space of the Analyzed GeneSet

  

Fig 3: GOMF\_PROTEIN\_PHOSPHATASE\_2A\_BINDING: Random ES distribution      
 Gene set null distribution of ES for **GOMF\_PROTEIN\_PHOSPHATASE\_2A\_BINDING**
